# Kidney Transplantation in Congenital Heart Disease Patients: What Are the Outcomes?

**DOI:** 10.1111/petr.70117

**Published:** 2025-06-09

**Authors:** Mario O'Connor, Joel T. Adler, Maria E. Hoyos, Michael D. Taylor, Neil M. Venardos, Carlos M. Mery, Charles D. Fraser, Andrew Well

**Affiliations:** ^1^ Texas Center for Pediatric and Congenital Heart Disease Dell Children's and UT Health Austin Texas USA; ^2^ Department of Surgery and Perioperative Care Dell Medical School at the University of Texas at Austin Austin Texas USA; ^3^ Division of Transplantation Dell Medical School at the University of Texas at Austin Austin Texas USA; ^4^ Department of Pediatrics Dell Medical School at the University of Texas at Austin Austin Texas USA; ^5^ Department of Surgery Oklahoma University Health Oklahoma City Oklahoma USA; ^6^ Department of Surgery Vanderbilt University Medical Center Nashville Tennessee USA

## Abstract

**Introduction:**

Congenital heart disease (CHD) patients experience risks for renal failure, including low cardiac output, exposure to nephrotoxic agents, and surgical interventions. Outcomes of kidney transplantation in CHD patients remain underexplored.

**Methods:**

A retrospective review of the Pediatric Health Information System database from 1/1/04–10/30/23. All patients < 18 years who underwent kidney transplantation were included. Multi‐organ transplants were excluded. International Classification of Diseases 9th and 10th editions were utilized to identify patients with a diagnosis consistent with CHD.

**Results:**

A total of 7586 patients were identified, with 3109 (41%) female, 3833 (51%) white non‐Hispanic, and a median age of 13 [IQR:7–16] years at transplant. A CHD diagnosis was present in 181 (2%). CHD patients were more likely to have mechanical ventilation > 96 h (*n* = 54 (30%) vs. *n* = 1264 (17%), *p* < 0.001) and red blood cell transfusion (*n* = 48 (27%) vs. *n* = 1452 (20%, *p* = 0.026)). On multivariable analysis, CHD was associated with a 9% (95% CI: 2.5–17.1) increase in length of stay (*p* = 0.007) and was not associated with an increased risk for 30‐day readmission (OR: 0.88; CI: 0.62–1.26, *p* = 0.509). Over a median follow‐up of 2.6 [IRQ: 0.7–5.7] years, no difference in re‐transplant free survival was evident.

**Conclusions:**

CHD patients have increased inhospital resource utilization compared to non‐CHD, but no difference in long‐term outcomes. However, differences in willingness to transplant cannot be assessed with this data. Further work is needed to better understand the impact of CHD on management decisions and outcomes across the kidney disease continuum.

## Graphical abstract

Patients with congenital heart disease (CHD) undergoing kidney transplant experience prolonged length of stay, increased risk for requiring prolonged mechanical ventilation, and posttransplant red blood cell transfusion. Overall, patients with CHD experienced similar long‐term outcomes.

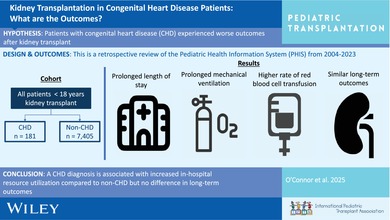

## 1 Introduction

Congenital heart disease (CHD) is the most common congenital malformation, with an incidence of 1% of live births [1]. With advances in CHD care, survival has significantly improved for CHD patients, with more than 97% reaching adolescence and adulthood [2]. Increased survival rate comes along with non‐cardiac complications related to the pathophysiology of their cardiac lesions and life‐time interventions [3].

End‐organ dysfunction has been identified as the new challenge in these patients, with a major impact on morbidity and mortality [4]. It has been reported that half of all adults living with CHD also have kidney disease [3]. Moreover, those who have kidney disease have a higher mortality rate compared to those with normal kidney function [5]. Reasons for this higher incidence of kidney dysfunction are complex and likely multifactorial and are broadly divided into intrinsic and extrinsic factors [6, 7].

Intrinsic factors depend on hemodynamic instability or degree of cyanosis, which correlates with heart lesion severity and/or heart function [6]. It has been noted that chronic hypoxia initially affects tubular function in the first decade of life, followed by altered glomerular filtration and leading to chronic kidney disease. Chronic hypoxia in CHD patients also stimulates erythropoietin production, increasing blood viscosity, altering renal hemodynamics, and reducing renal perfusion by increasing glomerular arteriolar resistance [7]. Extrinsic factors are those associated with congenital heart surgery, such as prolonged cardiopulmonary bypass time, prolonged cross‐clamp time, hypothermia, postoperative critical care management, and nephrotoxic agents [8, 9, 10].

Renal replacement therapies are widely used and have been well studied in the CHD population. Nonetheless, kidney transplant is still the gold standard treatment for end‐stage renal disease [9]. However, the incidence of and patient outcomes after kidney transplantation in CHD patients remain unexplored. This study aims to compare postoperative outcomes after renal transplantation in CHD patients compared to non‐CHD by utilizing a nation‐wide database.

## 2 Materials and Methods

Approval for this study was obtained after review by the Institutional Review Board for Dell Medical School at The University of Texas at Austin (STUDY00001853).

### 2.1 Data Source

This is a retrospective review using the Pediatric Health Information System (PHIS) database, from January 1, 2004, to October 30, 2023. PHIS, maintained by the Children's Hospital Association (CHA), encompasses administrative and billing data from forty‐nine pediatric hospitals, covering around 20% of pediatric hospitalizations nationwide. Of the 49 hospitals, 37 have an active pediatric kidney transplant program and actively contribute to PHIS.

Data is de‐identified upon submission and undergoes rigorous quality checks. Each patient is assigned a unique identifier for every hospital visit, enabling longitudinal tracking within the same facility. This includes not only inpatient encounters, but also ambulatory procedure visits, emergency department visits, and clinic visits. However, patients cannot be tracked across different CHA hospitals, as identifiers are unique to each individual facility. PHIS records include primary diagnoses, up to 41 additional diagnoses, principal procedures, and up to 41 additional procedures. For each procedure, PHIS also provides the specific hospital day on which it occurred relative to the admission date, allowing determination of the temporal sequence of procedures. Diagnoses and procedures were coded using the standard ICD, Ninth Revision (ICD‐9) until the third quarter of 2015. Records from the fourth quarter of 2015 through present were coded using the Tenth Revision (ICD‐10).

### 2.2 Study Population

All patients < 18 years of age in the PHIS database with an ICD code (ICD‐9: 55.69, ICD‐10: 0TY00Z0, 0TY10Z0) consistent with kidney transplant were included in the main cohort. Previously validated ICD codes were utilized to identify patients with a CHD diagnosis or diagnoses [10]. Those with a diagnosis consisting of isolated atrial septal defect (ASD) were not considered as CHD, as this code has been shown to have poor accuracy in administrative datasets [11]. Patients with isolated patent ductus arteriosus (PDA) were also considered non‐CHD patients. The following were excluded from analysis: events with missing information on the type of admission, sex, age, race, ethnicity, length of stay (LOS), discharge status, admitting diagnosis, and/or principal diagnosis.

The data presented in this study spans a 19‐year period and was divided into three evenly distributed eras: 2004–2009, 2010–2015, and 2016–2023. The most recent era (2016–2023) includes more years to account for the impact of the COVID‐19 pandemic, as prior studies have demonstrated a decline in the number of transplants performed during this time. The United States territory was divided into 4 different census regions, provided by PHIS: Midwest, Northeast, South, and West. Center volume was categorized into tertiles based on the median number of kidney transplant cases (submitted to PHIS) performed at each hospital per year, dividing them evenly into low tertile, middle tertile, or top tertile.

### 2.3 Study Outcomes

The primary aim of the study was to assess the incidence and outcomes of kidney transplant in patients with CHD. Demographics collected included age, sex, race, ethnicity, and insurance type. Insurance was grouped into Government (Medicare, Medicaid, Tricare, and CHIP), Private, and Other (Charity, other payor, private payers, and unknown). Hospitalization outcomes included length of stay (LOS) and in‐hospital mortality, all provided by PHIS. LOS was further subdivided into pre‐operative and postoperative LOS. Pre‐operative LOS was defined as the number of days from hospital admission to the date of the kidney transplant, while postoperative LOS represented the number of days from the transplant to hospital discharge. This stratification allowed for a more detailed evaluation of the clinical status and intensity of care between patient groups.

Additionally, the requirement for posttransplant hemodialysis, posttransplant prolonged invasive ventilatory support (defined as > 96 h), and posttransplant red blood cell transfusion (RBCT) was identified by ICD9/10 codes.

Patients were tracked for 30 days to evaluate the 30‐day readmission rate. Readmissions were classified into three categories: transplant‐related, infection‐related, and other causes. Transplant‐related causes encompassed rejection, surgical site complications, and reinterventions. Infection‐related causes included upper respiratory infections, gastrointestinal infections, and sepsis. Other causes referred to cases with no kidney transplant‐related surgical or diagnostic interventions.

Finally, patients who were alive at discharge were longitudinally followed within the same PHIS hospital using their unique identifier to assess post‐discharge survival and freedom from re‐transplantation. Follow‐up included all points of contact with the hospital, such as emergency department visits, observation unit stays, and outpatient clinic visits.

### 2.4 Statistical Analysis

Descriptive statistics were reported for demographics, clinical characteristics, and outcomes. Categorical variables are reported as n(%). LOS is reported in median [Interquartile Range (IQR)] days. Chi‐square and Fisher's exact test were utilized to analyze non‐continuous variables, as indicated. Kruskal‐Wallis test was utilized to analyze LOS comparison between groups. Multivariable analyses including linear and logistic multivariable regression were utilized to assess associations with CHD and outcomes. Cox regression analysis and Kaplan–Meier curves were utilized. All statistical tests were 2‐tailed and a *p*‐value < 0.05 was considered significant. Statistical analyses were performed using R and RStudio [12].

## 3 Results

### 3.1 Study Population and Demographics

From January 1, 2004, through October 30, 2023, there were 7586 patients that underwent kidney transplant. Of this cohort, 3109 (41%) were female, 3833 (51%) White Non‐Hispanic, 2292 (30%) had private insurance, and a median age of 13.0 [IQR:7.0–16.0] years at transplant. A total of 181 (2%) patients were identified as having a diagnosis consistent with CHD at any point in their lifetime. The 3 most common lesions were ventricular septal defect (VSD) (*n* = 47, 25.9%), congenital aortic stenosis (*n* = 36, 19.8%), and coarctation of the aorta (*n* = 32, 17.6%) (Table 2). CHD patients were younger, with a median age of 9.0 [IQR: 3.0–14.0] years at the time of transplant compared to non‐CHD patients, with a median age of 13.0 [IQR: 7.0–16.0] years at the time of transplant (*p* < 0.001). There were no significant differences in sex or race/ethnicity distribution between CHD and non‐CHD groups. The CHD group had more patients with private insurance compared to the non‐CHD group (*n* = 72 (40%) vs. *n* = 2220 (30%)) (Table 1).

**TABLE 1:** Demographics and clinical characteristics.

Variable	Overall (*n* = 7586)	CHD (*n* = 181) (2%)	Non‐CHD (*n* = 7405) (98%)	*p*
Age at Transplant (Years), Median [IQR]	13.0 [7.0–16.0]	9.0 [3.0–14.0]	13.0 [7.0–16.0]	**< 0.001**
Sex (*n*, %)				
Female	3109 (41)	63 (35)	3046 (41)	0.102
Race (*n*, %)				
White non‐Hispanic	3833 (51)	102 (56)	3731 (50)	0.466
Hispanic	1692 (22)	35 (19)	1657 (22)	
Black	1247 (16)	27 (15)	1220 (16)	
Other	814 (11)	17 (9)	797 (11)	
Insurance (*n*, %)				
Private	2292 (30)	72 (40)	2220 (30)	**0.014**
Government	3921 (52)	84 (46)	3837 (52)	
Other	1373 (18)	25 (14)	1348 (18)	
Region (*n*, %)				
Midwest	1965 (26)	50 (28)	1915 (26)	0.801
Northeast	1272 (17)	26 (14)	1246 (17)	
South	2285 (30)	57 (31)	2228 (30)	
West	2064 (27)	48 (27)	2016 (27)	
Era (*n*, %)				
2004–2009	1941 (26)	34 (2)	1907 (98)	**0.032**
2010–2015	2289 (30)	51 (2)	2238 (98)	
2016–2023	3356 (44)	96 (3)	3260 (97)	
Center volume (*n*, %)				
Top tertile	4813 (63)	102 (56)	4711 (64)	**0.006**
Middle tertile	2058 (27)	50 (28)	2008 (27)	
Bottom tertile	715 (9)	29 (16)	686 (9)	

*Note:* Bold values represent statistical significance.

No significant difference in the number of transplants was evident between census regions (*p* = 0.801). Most kidney transplants for the CHD group occurred between 2016 and 2023, accounting for over half of the total transplants in this group (*n* = 96 (53%)). (Table 1). Of all transplants, between 2004 and 2009, 34 (2%) kidney transplants were performed in patients with CHD, 51 (2%) between 2010 and 2015, and 96 (3%) between 2016 and 2023. The top tertile centers performed a median of 19.5 [15.3–21.0] surgical cases per year. Middle tertile centers carried out a median of 8.7 [7.6–9.3] cases per year, while bottom tertile centers had a median of 2 [1.2–5.2] cases per year (Table 1).

Of note, 22 (0.2%) patients underwent orthotopic heart transplantation before kidney transplant. Only 3 (14%) patients had a diagnosis consistent with CHD, including one patient with Ebstein anomaly, one patient with double outlet right ventricle, and a patient with hypoplastic left heart syndrome. Ten (45%) patients had a diagnosis of unspecified cardiomyopathy, 3 (14%) patients had medication‐induced cardiomyopathy, and 6 (27%) patients had myocarditis.

### 3.2 Outcomes

#### 3.2.1 Length of Stay

Overall, total median LOS at transplant was 9 [7–13] days, with the CHD group having longer LOS when compared to the non‐CHD group (10.0 [8.0–15.0] days vs. 9 [7.0–13.0], *p* < 0.001) (Table 2). Median postoperative LOS was 8.0 [6.0–12.0] days, with the CHD group having longer postoperative LOS compared to the non‐CHD group (9.0 [7.0–14.0] days vs. 8.0 [6.0–12.0] days, *p* < 0.001) (Table 2). After adjusting for sex, race, age, insurance type, center volume, census region, era, prolonged mechanical ventilation, RBCT, and need for posttransplant hemodialysis, CHD was associated with a 9.56% (95% CI: 2.52–17.09) increase in total LOS (*p* = 0.007; Table S1).

**TABLE 2:** Congenital heart disease diagnoses.

Congenital heart disease diagnosis	*n*/%
Ventricular septal defect	47 (25.9)
Congenital aortic stenosis	36 (19.8)
Coarctation of the aorta	32 (17.6)
Pulmonary artery stenosis (PA stenosis)	15 (8.2)
Atrial septal defect + ventricular septal defect	10 (5.5)
Pulmonary artery anomaly	8 (4.4)
Supra‐aortic stenosis	7 (3.8)
Single ventricle lesions	4 (2.2)
Tetralogy of Fallot	4 (2.2)
Interrupted aortic arch	3 (1.1)
Partial anomalous pulmonary venous return	3 (1.1)
Sub‐aortic stenosis	3 (1.1)
Atrioventricular septal defect	2 (11)
Double outlet right ventricle	2 (1.1)
Ebstein anomaly	2 (1.1)
Pulmonary atresia	2 (1.1)
Transposition of the great arteries	1 (0.5)

#### 3.2.2 Prolonged Mechanical Ventilation

**FIGURE 1:**
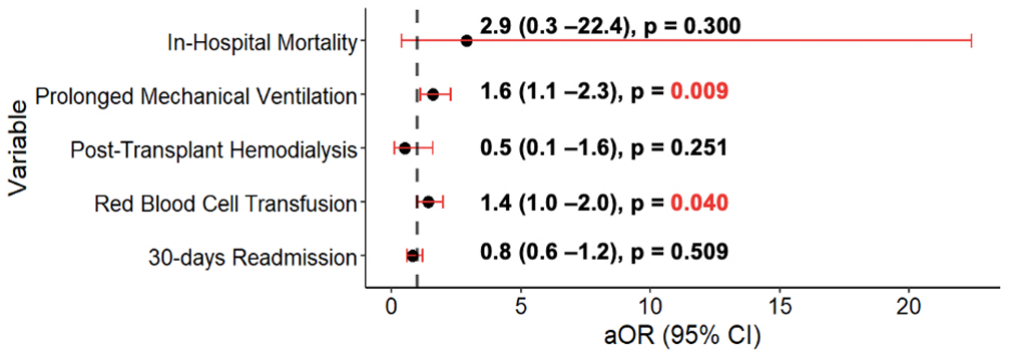
Multivariable logistic regression model results for CHD outcomes compared to non‐CHD. Adjusted for age, race, gender, insurance, era, center volume. aOR: adjusted odds ratio.

A total of 1318 (17%) patients needed prolonged mechanical ventilation. Of those, 54 (30%) of CHD patients needed prolonged mechanical ventilation compared to 1264 (17%) in the non‐CHD group (*p* < 0.001) (Table 3). After adjusting for sex, age, race, insurance, census region, era, and hospital tertile, CHD patients had higher odds of needing prolonged mechanical ventilation (OR: 1.60; 95% CI: 1.12–2.30, *p* = 0.009) (Figure 1; Table S2).

**TABLE 3:** Inhospital outcomes.

Variables	Overall (*n* = 7586)	CHD (*n* = 181) (2%)	Non‐CHD (*n* = 7405) (98%)	*p*
Length of stay (Days), Median [IQR]	9 [7–13]	10 [8–15]	9 [7–13]	**< 0.001**
Pre‐operative LOS (Days), Median [IQR]	1 [0–1]	1 [0–1]	1 [0–1]	0.059
Post‐operative LOS )(Days, Median [IQR]	8 [6–12]	9 [7–14]	8 [6–12]	**< 0.001**
Posttransplant prolonged ventilation	1318 (17)	54 (30)	1264 (17)	**< 0.001**
Posttransplant red blood cell transfusion	1500 (20)	48 (27)	1452 (20)	**0.026**
Posttransplant hemodialysis	239 (3)	3 (2)	236 (3)	0.342
Inhospital mortality	15 (0.2)	1 (0.5)	14 (0.1)	0.304

*Note:* Bold values represent statistical significance.

#### 3.2.3 Posttransplant Hemodialysis During Transplant Admission

A total of 239 (3%) patients required posttransplant hemodialysis during the transplant admission in the postoperative period. Median time to hemodialysis was 4.0 [2.0–7] days. Three (2%) patients in the CHD group and 236 (3%) in the non‐CHD group required hemodialysis; this was not different between groups (*p* = 0.342) (Table 3). In multivariable analysis, a CHD diagnosis (OR: 0.50; 95% CI: 0.15–1.61, *p* = 0.251) was not associated with increased risk of requiring hemodialysis posttransplant (Figure 1; Table S3).

#### 3.2.4 Posttransplant Red Blood Cell Transfusion

A total of 1500 (20%) patients required RBCT posttransplant. The CHD group was more likely to require RBCT compared to the non‐CHD group (*n* = 48 (27%) vs. *n* = 1452 (20%), *p* = 0.026). In multivariable analysis, CHD patients had an increased risk of having RBCT (OR: 1.44; 95% CI: 1.01–2.06, *p* = 0.040) (Table S4).

#### 3.2.5 Mortality

There were 15 (0.2%) in‐hospital mortalities; out of those, 1 (0.5%) occurred in the CHD group compared to 14 (0.1%) in the non‐CHD group (Table 2). In the univariate logistic regression model, CHD did not confer (OR: 2.93 CI: 0.38–22.42, *p* = 0.300) a significant increase in the odds for in‐hospital mortality (Figure 1) (Table S5).

#### 3.2.6 30‐Day Readmission

There was a total of 1831 readmission encounters within 30 days of discharge. Overall, the median number of readmissions per patient within 30 days was 1.0 [1.0–2.0] and this was not different between groups (*p* = 0.541). The most common reason for readmission was “other” (*n* = 797, 44%). CHD patients' most common reason was infectious related (*n* = 16, 37%) and “other” for the non‐CHD group (*n* = 784, 44%). There was a total of 1752 (23.1%) patients who had at least one readmission within 30 days of discharge. Forty‐one (22.7%) patients from the CHD group had at least one readmission within 30 days, compared to 1711 (23.1%) patients in the non‐CHD group. This was not different between groups (*p* = 0.957). The most common reason for readmission in the CHD group was infection related with 15 (37%), compared to the non‐CHD group where “other” was the most common cause (*n* = 750 (44%)) (Table 4). In multivariable analysis a CHD diagnosis (OR: 0.88; 95% CI: 0.62–1.26, *p* = 0.509) was still not associated with increased odds for 30‐day readmission (Figure 1) (Table S6).

**TABLE 4:** Episodes and reasons for 30‐day readmissions.

Reason for readmission	Overall (*n* = 1831)	CHD (*n* = 43) (2%)	Non‐CHD (*n* = 1788) (98%)	*p*
Transplant related	567 (31)	14 (33)	553 (29)	0.198
Infection related	467 (26)	16 (37)	451 (25)	
Other	797 (44)	13 (30)	784 (44)	

### 3.3 Long‐Term Outcomes

#### 3.3.1 Survival

Over a median follow‐up time of 2.7 [IQR: 0.7–5.7] years, a total of 87 patients died. The median time from transplant to death was 2.4 [IQR: 0.4–5.6]. Five (3%) patients died in the CHD group and 84 (1%) in the non‐CHD group, and this was not statistically significantly different between groups (*p* = 0.061) (Table 3). In univariate Cox regression analysis, a CHD diagnosis was not associated with increased mortality (HR: 1.91; 95% CI: 0.77–4.73, *p* = 0.161).

**FIGURE 2:**
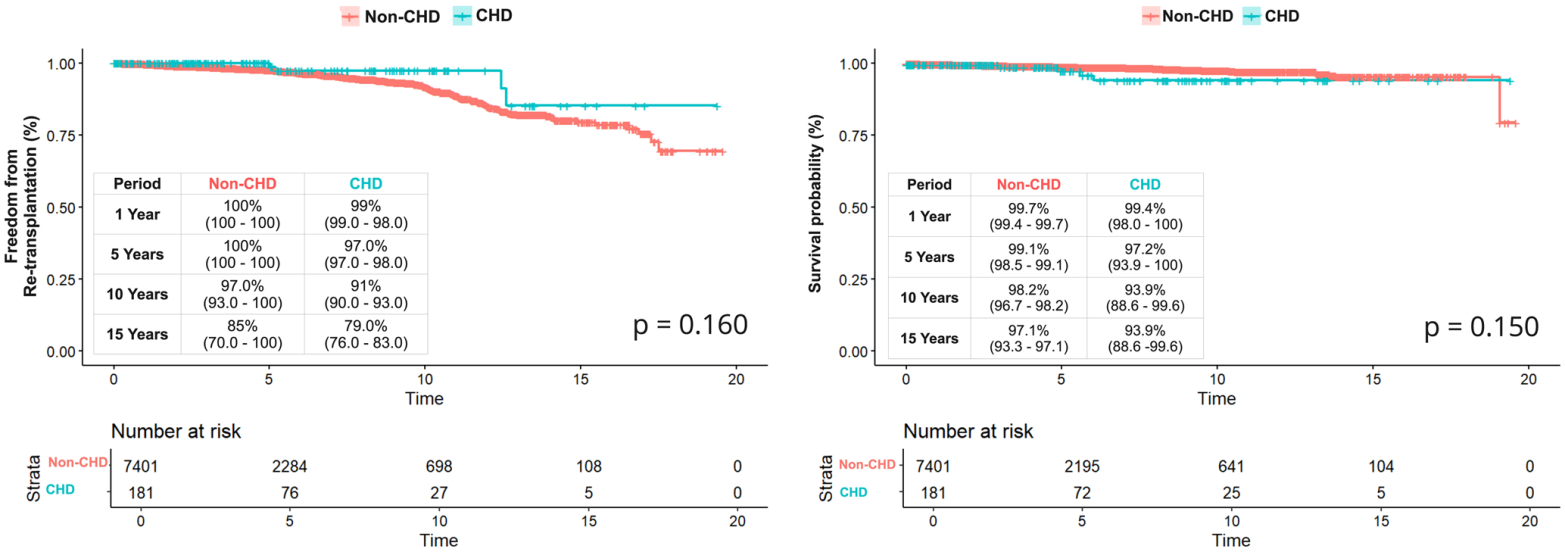
Freedom from re‐transplantation and survival.

When comparing patient survival using the Kaplan–Meier method, patient survival was not statistically significantly different between groups (log‐rank *p* = 0.150). The 1‐and 15‐year patient survival in the CHD group versus non‐CHD group was 99.4% versus 99.8% and 93.9% versus 95.2%, respectively (Figure 2).

#### 3.3.2 Re‐Transplantation

During the study period, 243 (3%) patients underwent re‐transplantation at the same initial transplanting facility. Of those, 4 (2%) patients were in the CHD group and 239 (3%) in the non‐CHD group. Median time to re‐transplantation was longer for CHD patients (105.7 [IQR: 61.7–149.8] months vs. 71.8 [IQR: 32.3–120.7] months, *p* < 0.001) (Table 5).

**TABLE 5:** Re‐transplantation.

Variables	Overall (*n* = 7586)	CHD (*n* = 181) (2%)	Non‐CHD (*n* = 7405) (98%)	*p*
Re‐transplantation	243 (3)	4 (2)	239 (3)	0.695
Median time in months to re‐transplantation in months [IQR]	71.5 [32.6–121.1]	105.7 [61.7–149.8]	71.8 [32.3–120.7]	**< 0.001**

*Note:* Bold values represent statistical significance.

In univariate Cox regression analysis, CHD patients did not have increased risk for re‐transplantation when compared to the non‐CHD group (HR: 0.49; 95% CI: 0.18–1.32, *p* = 0.162). In multivariable Cox regression analysis, a CHD diagnosis remained a nonsignificant factor for risk for re‐transplantation (HR: 0.59; 95% CI: 0.21–1.60, *p* = 0.305) (Table 6).

**TABLE 6:** Univariate/multivariable cox regression retransplantation.

Variable	Univariate			Mutivariable		
	HR	95% CI	*p*	HR	95% CI	*p*
Congenital heart disease	0.49	0.18–1.32	0.162	0.59	0.21–1.60	0.305
Age						
Age in years	1.13	1.11–1.47	**< 0.001**	1.09	1.07–1.11	**< 0.001**
Sex						
Female	0.92	0.71–1.20	0.566	0.89	0.68–1.16	0.401
Race						
White non‐Hispanic	**Ref**	**Ref**	**Ref**	**Ref**	**Ref**	**Ref**
Hispanic	1.11	0.81–1.52	0.486	0.97	0.69–1.36	0.876
Black	0.90	0.62–1.31	0.616	0.71	0.48–1.05	0.090
Other	0.76	0.46–1.23	0.266	0.78	0.48–1.26	0.340
Insurance						
Private	**Ref**	**Ref**	**Ref**	**Ref**	**Ref**	**Ref**
Government	0.91	0.69–1.20	0.508	1.21	0.91–1.62	0.184
Other	0.18	0.10–0.31	**< 0.001**	0.89	0.48–1.67	0.733
Region						
Midwest	0.91	0.64–1.32	0.658	0.92	0.62–1.36	0.700
Northeast	1.65	1.13–2.40	**0.008**	1.69	1.13–2.52	**0.009**
South	**Ref**	**Ref**	**Ref**	**Ref**	**Ref**	**Ref**
West	1.34	0.96–1.89	0.084	1.18	0.81–1.71	0.368
Era						
2004–2009	**Ref**	**Ref**	**Ref**	**Ref**	**Ref**	**Ref**
2010–2015	7.11	4.23–11.95	**< 0.001**	5.57	3.13–9.92	**< 0.001**
2016–2023	31.73	19.37–51.99	**< 0.001**	15.97	9.04–28.19	**< 0.001**
Center volume						
Top tertile	**Ref**	**Ref**	**Ref**	**Ref**	**Ref**	**Ref**
Middle tertile	0.92	0.69–1.23	0.594	0.99	0.72–1.36	0.973
Bottom tertile	0.54	0.30–0.98	**0.045**	0.59	0.32–1.10	0.099
Posttransplant red blood cell transfusion	0.57	0.41–0.80	**0.001**	1.10	0.77–1.56	0.585
Posttransplant hemodialysis	2.45	1.49–4.01	**< 0.001**	2.68	1.61–4.46	**< 0.001**

*Note:* Bold values represent statistical significance.

When comparing freedom from re‐transplantation using the Kaplan–Meier method, freedom from re‐transplantation was not statistically significantly different between groups (log‐rank *p* = 0.160). The 1‐ and 15‐year freedom from re‐transplantation in the CHD group versus non‐CHD group was 99% versus 100% and 79% versus 85%, respectively (Figure 2).

## 4 Discussion

This study utilizes data from the Pediatric Health Information System (PHIS) over 19 years to investigate incidence, short‐term outcomes, and long‐term outcomes of kidney transplantation in CHD patients. Among the study cohort, 181 (2%) patients with a CHD diagnosis underwent kidney transplantation. Overall, our study found that CHD patients have increased in‐hospital resource utilization compared to non‐CHD patients, with prolonged length of stay and mechanical ventilation, but no observable impact on mortality and long‐term outcomes.

It is known that patients with CHD are at increased risk for chronic kidney disease (CKD) due to several factors associated with their cardiac condition, such as hypoperfusion, chronic hypoxia, and altered hemodynamics [3, 13, 14]. On the other hand, repetitive exposure to nephrotoxic agents such as iodinated contrast for computer tomography scans and cardiac catheterizations increases their lifetime risk [15]. As well, CHD patients could undergo surgical procedures that may involve the use of a cardiopulmonary bypass machine, and although advancements in this area have occurred, kidney injury is still a common complication after cardiac surgery [13]. Although external insults contribute significantly to the increased risk of renal disease in patients with CHD, it is also well recognized that developmental abnormalities of the kidney are prevalent within this population [16]. Approximately 20% to 30% of CHD patients present with congenital or developmental kidney abnormalities, which can independently lead to the development of end‐stage renal disease (ESRD) [17]. These abnormalities may include structural anomalies such as renal hypoplasia, dysplasia, or anomalies in renal vasculature [18].

Inhospital outcomes were good for the entire cohort. Of note, there were only 15 in‐hospital mortalities with only 1 in the CHD group. Having a diagnosis consistent with CHD did not confer an increased risk for in‐hospital mortality. The overall median length of stay was 9 [IQR: 9–13] days, which is in line with other published pediatric reports [19, 20]. CHD patients were found to have longer median lengths of stay, and this difference remains after adjustment for potential confounders, with CHD conferring a 10% increase in length of stay. CHD patients had a higher risk of prolonged mechanical ventilation, and this risk remains more than double after adjusting for confounders.

The factors contributing to an extended length of hospital stay (LOS) and prolonged mechanical ventilation in patients with congenital heart disease (CHD) require further examination. Investigating the reasons behind the prolonged postoperative period in CHD patients is crucial to identify if it is either linked to the transplant, surgical complications, postoperative cardiac assessments, or hospital protocols. Further evaluation is needed to determine if CHD patients genuinely require an extended stay or if other non‐medical factors are limiting their discharge and its impact on resource utilization.

In our cohort, CHD patients were more likely to receive a blood transfusion when compared to non‐CHD patients. Given the risk of sensitization with blood transfusions and past associations with delayed graft function and bacterial infection, it is recommended to follow a restrictive transfusion practice in patients undergoing kidney transplant [21, 22, 23, 24]. Given this, it is crucial to investigate the underlying reasons for transfusing these patients and whether a less permissive anemia is adopted due to the cardiac or physiologic condition.

Looking at 30‐day readmissions, 23% of our cohort had at least 1 readmission during the first 30 days post‐discharge. National baseline readmission rates are not available for pediatric transplant programs; thus, we have no method of comparison. That being said, single center experiences have reported between 20% and 50% readmission rates within the first 30 days postdischarge. This compares with adult programs where the rate of readmission is between 15% and 35% [25, 26]. No difference in readmission rates between CHD and non‐CHD patients was evident in our cohort [25, 27, 28]. Further studies are needed to evaluate the level of care associated with these readmissions and to determine if CHD patients require a higher level of care.

Due to ongoing research, there have been significant advancements in both graft survival and the long‐term survival of pediatric patients with ESRD over time [29, 30]. In our study, over a median time of 2.7 [IQR: 0.7–5.7] years, no difference in re‐transplantation rates between CHD and non‐CHD was found. Freedom from re‐transplantation at 15 years was 79% in the CHD group and 85% in the non‐CHD group. Factors associated with an increased risk of re‐transplantation included older age at transplant and the need for posttransplant hemodialysis. These results differ from previous reports where kidney graft survival at 15 years in the normal pediatric population is reported between 65% and 75% [29, 31] We recognize that graft loss rates are higher, and the data presented reflect an optimistic scenario. However, our capacity to identify patients who are dependent on dialysis and the limitations imposed by re‐transplantation at other facilities contribute to reporting more favorable outcomes in this analysis.

### 4.1 Limitations

The limitations of this study should be noted. As it relies on an administrative dataset, all inherent limitations, including the potential for erroneous coding of diagnosis and procedure codes, are present, with a higher risk due to the complexities of CHD. The lack of detailed clinical and donor data limits the development of risk profiles. Furthermore, the cohort includes only patients from free‐standing children's hospitals, excluding those from non‐children's hospitals performing pediatric kidney transplants, potentially underreporting CHD patients undergoing kidney transplants and may also result in differing graft survival rates compared to other reports. Furthermore, the ability to track patients with graft failure on hemodialysis is limited because those receiving care at outside facilities are not captured, potentially leading to an overreporting of graft survival. Finally, survival analysis in this study represents the best‐case scenario as mortalities outside a PHIS facility are not recorded.

## 5 Conclusions

CHD patients undergoing kidney transplantation have higher in‐hospital resource utilization, including longer hospital stays and increased need for mechanical ventilation and blood transfusions. Despite these differences, long‐term outcomes such as 30‐day readmission rates, survival, and re‐transplantation do not show significant variability between CHD and non‐CHD patients. Further research is necessary to explore the impact of CHD on clinical decision‐making and long‐term management of kidney transplant recipients across the continuum of kidney disease.

## Supporting information


Tables S1‐S6.

## Data Availability

The data that support the findings of this study are available from the corresponding author upon reasonable request.
