# Supplementary material for: Kidney Transplantation in Congenital Heart Disease Patients: What Are the Outcomes?
Source: Pediatr Transplant. 2025 Jun 9;29(5):e70117. doi: 10.1111/petr.70117 (PMC12147195; doi:10.1111/petr.70117)
Supplement: Supplementary file 1 — Tables S1‐S6. [file PETR-29-e70117-s001.docx]

Supplement

Table S1. Multivariable Linear Model Length of Stay

| Variable | Percent difference,% | 95% CI | p-value |
| --- | --- | --- | --- |
| Congenital Heart Disease | 9.56 | 2.52 – 17.09 | **0.007** |
|  |  |  |  |
| Age |  |  |  |
| Age in Years | -1.97 | -2.13 – 1.81 | **<0.001** |
| Sex |  |  |  |
| Female | 1.15 | -0.90 – 3.26 | 0.273 |
| Race |  |  |  |
| White non-Hispanic | Ref | Ref | Ref |
| Hispanic | 3.03 | 0.24 – 5.91 | **0.033** |
| Black | 7.74 | 4.56 – 11.01 | **<0.001** |
| Other | 2.91 | -0.56 – 6.51 | 0.102 |
| Insurance |  |  |  |
| Private | Ref | Ref | Ref |
| Government | 10.59 | 7.97 – 13.28 | **<0.001** |
| Other | 3.30 | -0.21 – 6.94 | 0.066 |
| Region |  |  |  |
| Midwest | 12.07 | 8.84 – 15.39 | **<0.001** |
| Northeast | 14.30 | 10.62 – 18.10 | **<0.001** |
| South | Ref | Ref | **Ref** |
| West | -3.45 | -6.29 – -0.53 | **0.020** |
| Era |  |  |  |
| 2004-2009 | Ref | Ref | Ref |
| 2010-2015 | -1.99 | -4.92 – 1.02 | 0.193 |
| 2016-2023 | 4.27 | 1.30 – 7.33 | **0.004** |
| Center volume |  |  |  |
| Top tertile | Ref | Ref | Ref |
| Middle tertile | -20.52 | -22.43 – -18.57 | **<0.001** |
| Bottom tertile | -12.16 | -15.46 – -8.73 | **<0.001** |
|  |  |  |  |
| Prolonged Ventilation | 38.24 | 34.36 – 42.23 | **<0.001** |
| Red Blood Cell Transfusion | 18.42 | 15.30 – 21.63 | **<0.001** |
| Post-transplant Hemodialysis | 74.13 | 64.27 – 84.60 | **<0.001** |

Table S2. Logistic Regression Model Prolonged Ventilation

|  | Univariate | | | Multivariate | | |
| --- | --- | --- | --- | --- | --- | --- |
| Variable | OR | 95% CI | p value | OR | 95% CI | p-value |
| Congenital Heart Disease | 2.06 | 1.49 – 2.85 | **<0.001** | 1.60 | 1.12 – 2.30 | **0.009** |
|  |  |  |  |  |  |  |
| Age |  |  |  |  |  |  |
| Age in Years | 0.86 | 0.85 – 0.87 | **<0.001** | 0.86 | 0.85 – 0.87 | **<0.001** |
| Sex |  |  |  |  |  |  |
| Female | 0.79 | 0.70 – 0.90 | **<0.001** | 0.87 | 0.76 – 1.00 | 0.057 |
| Race |  |  |  |  |  |  |
| White non-Hispanic | Ref | Ref | Ref | Ref | Ref | Ref |
| Hispanic | 1.11 | 0.96 – 1.29 | 0.141 | 1.18 | 0.99 – 1.40 | 0.058 |
| Black | 1.00 | 0.84 – 1.18 | 0.989 | 1.29 | 1.06 – 1.56 | **0.009** |
| Other | 1.01 | 0.83 – 1.24 | 0.881 | 1.03 | 0.83 – 1.28 | 0.762 |
| Insurance |  |  |  |  |  |  |
| Private | Ref | Ref | Ref | Ref | Ref | Ref |
| Government | 1.16 | 1.01 – 1.34 | **0.031** | 1.03 | 0.88 – 1.20 | 0.680 |
| Other | 1.33 | 1.11 – 1.58 | **0.001** | 0.97 | 0.78 – 1.21 | 0.825 |
| Region |  |  |  |  |  |  |
| Midwest | 1.01 | 0.86 – 1.20 | 0.827 | 0.86 | 0.71 – 1.05 | 0.149 |
| Northeast | 1.01 | 0.84 – 1.22 | 0.857 | 0.91 | 0.73 – 1.13 | 0.409 |
| South | Ref | Ref | **Ref** | Ref | Ref | **Ref** |
| West | 1.40 | 1.20 – 1.64 | **<0.001** | 1.62 | 1.34 – 1.96 | <0.001 |
| Era |  |  |  |  |  |  |
| 2004-2009 | Ref | Ref | Ref | Ref | Ref | Ref |
| 2010-2015 | 0.69 | 0.59 – 0.80 | **<0.001** | 0.60 | 0.50 – 0.73 | <0.001 |
| 2016-2023 | 0.56 | 0.49 – 0.65 | **<0.001** | 0.55 | 0.46 – 0.66 | **<0.001** |
| Center volume |  |  |  |  |  |  |
| Top tertile | Ref | Ref | Ref | Ref | Ref | Ref |
| Middle tertile | 0.94 | 0.82 – 1.08 | 0.405 | 1.10 | 0.94 – 1.29 | 0.190 |
| Bottom tertile | 0.89 | 0.72 – 1.10 | 0.319 | 1.09 | 0.85 – 1.41 | 0.467 |

Table S3. Logistic Regression Model Post-transplant HD

|  | Univariate | | | Multivariate | | |
| --- | --- | --- | --- | --- | --- | --- |
| Variable | OR | 95% CI | p value | OR | 95% CI | p-value |
| Congenital Heart Disease | 0.51 | 0.16 – 1.61 | 0.253 | 0.50 | 0.15 – 1.61 | 0.251 |
|  |  |  |  |  |  |  |
| Age |  |  |  |  |  |  |
| Age in Years | 1.01 | 0.99 – 1.03 | 0.214 | 1.02 | 1.00 – 1.04 | **0.005** |
| Sex |  |  |  |  |  |  |
| Female | 0.96 | 0.74 – 1.25 | 0.794 | 0.95 | 0.73 – 1.24 | 0.749 |
| Race |  |  |  |  |  |  |
| White non-Hispanic | **Ref** | **Ref** | **Ref** | **Ref** | **Ref** | **Ref** |
| Hispanic | 0.88 | 0.62 – 1.25 | 0.486 | 0.88 | 0.60 – 1.28 | 0.512 |
| Black | 1.60 | 1.16 – 2.21 | **0.003** | 1.51 | 1.08 – 2.13 | 0.015 |
| Other | 0.81 | 0.50 – 1.31 | 0.402 | 0.84 | 0.51 – 1.37 | 0.489 |
| Insurance |  |  |  |  |  |  |
| Private | **Ref** | **Ref** | **Ref** | **Ref** | **Ref** | **Ref** |
| Government | 1.50 | 1.09 – 2.07 | **0.012** | 1.42 | 1.01 – 1.99 | **0.038** |
| Other | 1.62 | 1.10 – 2.40 | **0.014** | 1.19 | 0.76 – 1.86 | **0.429** |
| Region |  |  |  |  |  |  |
| Midwest | 1.39 | 0.99 – 1.94 | 0.053 | 1.47 | 1.02 – 2.14 | **0.038** |
| Northeast | 1.31 | 0.89 – 1.91 | 0.165 | 1.57 | 1.03 – 2.38 | **0.033** |
| South | **Ref** | **Ref** | **Ref** | **Ref** | **Ref** | **Ref** |
| West | 0.84 | 0.58 – 1.23 | 0.386 | 1.12 | 0.73 – 1.72 | 0.586 |
| Era |  |  |  |  |  |  |
| 2004-2009 | **Ref** | **Ref** | **Ref** | **Ref** | **Ref** | **Ref** |
| 2010-2015 | 0.71 | 0.52 – 0.98 | **0.042** | 0.75 | 0.53 – 1.08 | 0.131 |
| 2016-2023 | 0.54 | 0.39 – 0.73 | **<0.001** | 0.66 | 0.46 – 0.94 | **0.022** |
| Center volume |  |  |  |  |  |  |
| Top tertile | **Ref** | **Ref** | **Ref** | **Ref** | **Ref** | **Ref** |
| Middle tertile | 1.05 | 0.78 – 1.41 | 0.728 | 1.08 | 0.79 – 1.48 | 0.589 |
| Bottom tertile | 1.04 | 0.66 – 1.62 | 0.862 | 1.30 | 0.79 – 2.14 | 0.284 |
|  |  |  |  |  |  |  |
| Red Blood Cell Transfusion | 3.00 | 2.30 – 3.90 | **<0.001** | 3.01 | 2.28 – 3.99 | **<0.001** |

Table S4. Univariate and Multivariate Logistic Regression Model Red Blood Cell Transfusion

|  | Univariate | | | Multivariate | | | |
| --- | --- | --- | --- | --- | --- | --- | --- |
| Variable | OR | 95% CI | P value | OR | 95% CI | p-value |  |
| Congenital Heart Disease | 1.47 | 1.05 – 2.06 | **0.021** | 1.44 | 1.01 –2.06 | **0.040** |  |
|  |  |  |  |  |  |  |  |
| Age |  |  |  |  |  |  |  |
| Age in Years | 0.92 | 0.91 – 0.93 | **<0.001** | 0.91 | 0.91 –0.92 | **<0.001** |  |
| Sex |  |  |  |  |  |  |  |
| Female | 0.97 | 0.87 – 1.09 | 0.629 | 1.08 | 0.95 –1.22 | 0.201 |  |
| Race |  |  |  |  |  |  |  |
| White non-Hispanic | **Ref** | **Ref** | **Ref** | **Ref** | **Ref** | **Ref** |  |
| Hispanic | 1.01 | 0.87 – 1.16 | 0.885 | 1.29 | 1.10 –1.52 | 0.001 |  |
| Black | 1.01 | 0.86 – 1.19 | 0.818 | 1.14 | 0.96 –1.36 | 0.121 |  |
| Other | 0.96 | 0.79 – 1.16 | 0.703 | 1.11 | 0.91 –1.37 | 0.284 |  |
| Insurance |  |  |  |  |  |  |  |
| Private | **Ref** | **Ref** | **Ref** | **Ref** | **Ref** | **Ref** |  |
| Government | 1.16 | 1.02 – 1.33 | **0.023** | 1.06 | 0.92 –1.23 | 0.370 |  |
| Other | 1.35 | 1.15 – 1.60 | **<0.001** | 0.76 | 0.62 –0.92 | **0.006** |  |
| Region |  |  |  |  |  |  |  |
| Midwest | 1.69 | 1.46 – 1.96 | **<0.001** | 1.55 | 1.32 –1.83 | **<0.001** |  |
| Northeast | 1.10 | 0.92 – 1.31 | 0.273 | 0.86 | 0.71 –1.04 | 0.141 |  |
| South | **Ref** | **Ref** | **Ref** | **Ref** | **Ref** | **Ref** |  |
| West | 0.83 | 0.71 – 0.98 | **0.027** | 0.66 | 0.55 –0.79 | **<0.001** |  |
| Era |  |  |  |  |  |  |  |
| 2004-2009 | **Ref** | **Ref** | **Ref** | **Ref** | **Ref** | **Ref** |  |
| 2010-2015 | 0.82 | 0.71 – 0.94 | **0.006** | 0.69 | 0.58 – 0.81 | **<0.001** |  |
| 2016-2023 | 0.39 | 0.34 – 0.45 | **<0.001** | 0.33 | 0.28 – 0.38 | **<0.001** |  |
| Center volume |  |  |  |  |  |  |  |
| Top tertile | **Ref** | **Ref** | **Ref** | **Ref** | **Ref** | **Ref** |  |
| Middle tertile | 0.91 | 0.80 – 1.04 | 0.188 | 0.83 | 0.72 – 0.96 | **0.012** |  |
| Bottom tertile | 0.52 | 0.41 – 0.65 | **<0.001** | 0.47 | 0.36 – 0.61 | **<0.001** |  |
|  |  |  |  |  |  |  |  |
| Post-transplant Hemodialysis | 3.00 | 2.30 – 3.90 | **<0.001** | 3.03 | 2.29 – 4.01 | **<0.001** |  |

Table S5. Univariate Logistic Regression Model Mortality

| Variable | OR | 95% CI | p-value |
| --- | --- | --- | --- |
| Congenital Heart Disease | 2.93 | 0.38-22.42 | 0.300 |
|  |  |  |  |
| Age |  |  |  |
| Age in Years | 0.73 | 0.63-0.85 | **<0.001** |
| Sex |  |  |  |
| Female | 1.64 | 0.59-4.54 | 0.335 |
| Race |  |  |  |
| White non-Hispanic | Ref | Ref | Ref |
| Hispanic | 1.00 | 0.30 – 3.27 | 0.991 |
| Black | 0.34 | 0.04 – 2.69 | 0.308 |
| Other | 0.52 | 0.06 – 4.13 | 0.538 |
| Insurance |  |  |  |
| Private | Ref | Ref | Ref |
| Government | 1.55 | 0.41 – 0.5.88 | 0.512 |
| Other | 2.22 | 0.49 – 9.97 | 0.294 |
| Region |  |  |  |
| South | Ref | Ref | Ref |
| Midwest | 4.08 | 0.84 – 19.66 | 0.079 |
| Northeast | 0.001 | 0.00 – >1000 | 0.985 |
| West | 3.32 | 0.67 – 16.50 | 0.141 |
| Era |  |  |  |
| 2004-2009 | Ref | Ref | Ref |
| 2010-2015 | 0.42 | 0.10 – 1.69 | 0.224 |
| 2016-2023 | 0.57 | 0.18 – 1.79 | 0.342 |
| Center volume |  |  |  |
| Top tertile | Ref | Ref | Ref |
| Middle tertile | 2.00 | 0.67 – 5.98 | 0.211 |
| Bottom tertile | 1.92 | 0.39 – 9.28 | 0.414 |
|  |  |  |  |
| Length of stay (days) Median [IQR] | 1.00 | 1.004 – 1.014 | **<0.001** |
| Pre-operative LOS (days), Median[IQR] | 1.01 | 1.005 – 1.021 | **0.001** |
| Post-operative LOS (days),Median [IQR] | 1.01 | 1.008 – 1.026 | **<0.001** |
| Prolonged Ventilation | >100 | 0.00 – >1000 | 0.985 |
| Red Blood Cell Transfusion | 2.03 | 0.69 – 5.95 | 0.196 |
| Post-transplant Hemodialysis | 7.77 | 2.17 – 27.71 | **0.001** |

Table S6. Logistic Regression Model 30d Readmission (Yes/No)

|  | Univariate | | | Multivariate | | | |
| --- | --- | --- | --- | --- | --- | --- | --- |
| Variable | OR | 95% CI | p value | OR | 95% CI | p-value |  |
| Congenital Heart Disease | 0.97 | 0.68 – 1.38 | 0.886 | 0.88 | 0.62 – 1.26 | 0.509 |  |
|  |  |  |  |  |  |  |  |
| Age |  |  |  |  |  |  |  |
| Age in Years | 0.96 | 0.96-0.97 | **<0.001** | 0.97 | 0.96 – 0.98 | **<0.001** |  |
| Sex |  |  |  |  |  |  |  |
| Female | 1.01 | 0.91 – 1.13 | 0.783 | 1.04 | 0.93 – 1.16 | 0.428 |  |
| Race |  |  |  |  |  |  |  |
| White non-Hispanic | **Ref** | **Ref** | **Ref** | **Ref** | **Ref** | **Ref** |  |
| Hispanic | 0.90 | 0.78 – 1.04 | 0.165 | 0.89 | 0.76 – 1.03 | 0.139 |  |
| Black | 1.10 | 0.95 – 1.28 | 0.175 | 1.12 | 0.96 -1.31 | 0.134 |  |
| Other | 1.14 | 0.95 – 1.35 | 0.141 | 1.15 | 0.96 – 1.38 | 0.110 |  |
| Insurance |  |  |  |  |  |  |  |
| Private | **Ref** | **Ref** | **Ref** | **Ref** | **Ref** | **Ref** |  |
| Government | 1.17 | 1.04 – 1.33 | **0.008** | 1.16 | 1.02 – 1.32 | **0.021** |  |
| Other | 1.04 | 0.88 – 1.22 | 0.631 | 0.95 | 0.79 – 1.14 | 0.612 |  |
| Region |  |  |  |  |  |  |  |
| Midwest | 1.10 | 0.95 – 1.27 | 0.175 | 1.11 | 0.95 – 1.30 | 0.165 |  |
| Northeast | 0.91 | 0.77 – 1.07 | 0.268 | 0.94 | 0.79 – 1.13 | 0.568 |  |
| South | **Ref** | **Ref** | **Ref** | **Ref** | **Ref** | **Ref** |  |
| West | 1.03 | 0.89 – 1.18 | 0.669 | 1.12 | 0.95 – 1.31 | 0.149 |  |
| Era |  |  |  |  |  |  |  |
| 2004-2009 | **Ref** | **Ref** | **Ref** | **Ref** | **Ref** | **Ref** |  |
| 2010-2015 | 1.03 | 0.90 – 1.19 | 0.590 | 0.98 | 0.84 – 1.15 | 0.849 |  |
| 2016-2023 | 0.83 | 0.72 – 0.94 | **0.006** | 0.80 | 0.68 – 0.93 | **0.004** |  |
| Center volume |  |  |  |  |  |  |  |
| Top tertile | **Ref** | **Ref** | **Ref** | **Ref** | **Ref** | **Ref** |  |
| Middle tertile | 1.17 | 0.97 – 1.40 | 0.089 | 1.22 | 1.07 – 1.39 | **0.001** |  |
| Bottom tertile | 1.20 | 1.06 – 1.35 | **0.002** | 1.20 | 0.98 – 1.47 | 0.070 |  |
|  |  |  |  |  |  |  |  |
| Prolonged Ventilation | 1.61 | 1.41 – 1.84 | **<0.001** | 1.38 | 1.20 – 1.59 | **<0.001** |  |
| Red Blood Cell Transfusion | 1.26 | 1.10 – 1.43 | **<0.001** | 1.05 | 0.92 – 1.21 | 0.429 |  |
| Post-transplant Hemodialysis | 1.45 | 1.09 – 1.92 | **0.009** | 1.32 | 0.99 – 1.76 | 0.054 |  |
